# Impaired Cav-1 expression in SSc mesenchymal cells upregulates VEGF signaling: a link between vascular involvement and fibrosis

**DOI:** 10.1186/1755-1536-7-13

**Published:** 2014-09-15

**Authors:** Paola Cipriani, Paola Di Benedetto, Daria Capece, Francesca Zazzeroni, Vasiliki Liakouli, Piero Ruscitti, Ilenia Pantano, Onorina Berardicurti, Francesco Carubbi, Edoardo Alesse, Roberto Giacomelli

**Affiliations:** 1 Department of Applied Clinical Sciences and Biotechnology, Rheumatology Unit, School of Medicine, University of L’Aquila, Delta 6 Building, Via dell’Ospedale, 67100 L’Aquila, Italy; 2 Department of Applied Clinical Sciences and Biotechnology, University of L’Aquila, Coppito 2, 67100 L’Aquila, Italy

**Keywords:** Systemic sclerosis, Mesenchymal stem cells, Pericytes, Caveolin-1

## Abstract

**Background:**

Systemic sclerosis (SSc) is characterized by vascular alteration and fibrosis, the former probably leading to fibrosis via the ability of both endothelial cells and pericytes to differentiate toward myofibroblast. It is well known that vascular endothelial growth factor A (VEGF-A, hereafter referred to as VEGF) may induce a profibrotic phenotype on perivascular cells. Caveolin-1 (Cav-1) is involved in the regulation of VEGF signaling, playing a role in the transport of internalized VEGF receptor 2 (VEGFR2) toward degradation, thus decreasing VEGF signaling. In this work, we assessed the levels of Cav-1 in SSc bone marrow mesenchymal stem cells (SSc-MSCs), a pericyte surrogate, and correlate these results with VEGF signaling, focusing onpotential pathogenic pathways leading to fibrosis.

**Results:**

We explored the VEGF signaling assessing: (1) Cav-1 expression; (2) its co-localization with VEGFR2; (3) the activity of VEGFR2, by IF, immunoprecipitation, and western blot. In SSc-MSCs, Cav-1 levels were lower when compared to healthy controls (HC)-MSCs. Furthermore, the Cav-1/VEGFR2 co-localization and the ubiquitination of VEGFR2 were impaired in SSc-MSCs, suggesting a decreased degradation of the receptor and, as a consequence, the tyrosine phosphorylation of VEGFR2 and the PI3-kinase-Akt pathways were significantly increased when compared to HC. Furthermore, an increased connective tissue growth factor (CTGF) expression was observed in SSc-MSCs. Taken together, these data suggested the upregulation of VEGF signaling in SSc-MSCs. Furthermore, after silencing Cav-1 expression in HC-MSCs, an increased CTGF expression in HC-MSCs was observed, mirroring the results obtained in SSc-MSCs, and confirming the potential role that the lack of Cav-1 may play in the persistent VEGF signaling .

**Conclusions:**

During SSc, the lower levels of Cav-1 may contribute to the pathogenesis of fibrosis via an upregulation of the VEGF signaling in perivascular cells which are shifted to a profibrotic phenotype.

## Background

Systemic sclerosis (SSc) is a chronic autoimmune connective tissue disease affecting the skin and a variety of internal organs. The hallmark of the early stages of SSc is endothelial involvement, with a perivascular inflammatory infiltrates and a decreased capillary density [1], whereas later stages are characterized by an excessive accumulation of extracellular matrix (ECM), resulting in fibrosis [2,3]. The vascular alterations might be considered an important trigger for fibrosis, because of endothelial cells and pericytes, after injuries, might trans-differentiate toward myofibroblast, a cell producing increased amounts of collagen types I, III, VI, and VII, fibronectin, and glycosaminoglycans [4].

Originally, myofibroblasts were thought to derive from resident fibroblasts which, after the interaction with effector molecules in injured tissues, might be activated, thus leading to reparative fibrotic response. More recently, it has been suggested that these cells may be generated by both epithelial-mesenchymal and endothelial-mesenchymal transformation [5-7]. In the last years, the use of lineage tracing, helped us to better define the myofibroblasts origin and evolution, confirming that pericytes and resident fibroblasts are the major, if not the only, source of myofibroblasts in at least one animal model of kidney fibrotic diseases [8-10]. On these bases, it might be important to assess if the molecular mechanisms involved in the differentiation of pericytes toward myofibroblasts may play a role in the pathological process leading to fibrosis during SSc.

Recent works showed that perivascular cells share surface markers and differentiative ability with bone marrow mesenchymal stem cells (MSCs), and MSCs express pericyte markers and cooperate with endothelial cells to form a vascular network, supporting the concept that pericytes are members of the adult multipotent MSCs family [11-15]. Because of these similarities and to overcome the difficulties to isolate primary pericytes, as reported by several authors [16,17], in our work we used MSCs as pericytes. Furthermore, we already reported that SSc-MSCs have been shown to be an alternative system for studying the contribution of pericytes in the pathogenesis of SSc [18].

Vascular endothelial growth factor A (VEGF) is a key growth factor, involved in the reparative angiogenesis, after injuries [19,20]. In addition to this well known effect, this molecule may induce perivascular cells to produce connective tissue growth factor (CTGF), which stimulates extracellular matrix production and fibrosis [21]. In fact, in presence of CTGF, pericytes differentiate toward a migratory and myofibroblast phenotype [22]. VEGF has been suggested to be involved in the pathogenesis of SSc and its expression is markedly increased in different cell types, both in the epidermis and dermis of patients with SSc and in the bloodstream, correlating with organ manifestations [23,24]. At present, many different isoforms of VEGF family are known, such as VEGF121, VEGF189, and VEGF165, and recently, a new VEGF165 isoform (VEGF165b), generated through alternative splicing, with a possible antiangiogenic effect, has been described [25]. Although a switch from proangiogenic to antiangiogenic isoforms has been recently described in SSc patients [26], available literature showed conflicting results about the potential inhibitory role of this isoform [25,27]. VEGF exerts its functions by binding to the tyrosine kinase receptors VEGF receptor 1 (VEGFR1) and VEGF receptor 2 (VEGFR2). The tridimensional VEGFR organization, on cell surface, modulates the transmission of its specific signal. It is well known that the cell surface proteins trafficking is regulated by the lipid rafts microdomains which are present in the plasma membranes. Caveolae are special structures of lipid raft, which may be detected by electron microscopy as flask shaped invaginations in the cell surface membrane [28,29]. These structures are enriched in cholesterol and may be distinguished from other structures of the lipid raft by the presence of a specific molecular family: the caveolin scaffolding proteins, which are essential for caveolae formation and cholesterol binding [30-32]. Caveolin-1 (Cav-1) is a member of this family and it is involved in the regulation of signaling activity, via a direct interaction of its scaffolding domain with a consensus sequence present in several signaling proteins, including VEGFR2 [33]. Of note, the interaction of Cav-1 with the signal transducing proteins, has been shown to inhibit the activity of the latters. On these bases, Cav-1 may play an important role in VEGF-induced signaling, promoting VEGFR2 degradation by caveolin-mediated transport to proteasome, under specific conditions.

Conflicting results have been published on available literature concerning Cav-1 in SSc. Lower levels of Cav-1 were found in SSc lung fibroblasts and linked to constitutive activation of JNK, ERK, and Akt signaling, leading to overexpression of profibrotic markers such as collagen and alpha smooth muscle actin [34]. Del Galdo *et al*. [35] showed, in dermal fibroblasts, a downregulation of Cav-1 in SSc skin and suggested that this finding may contribute to the increased collagen deposition via the activation of canonical transforming growth factor (TGF-b) pathway. Haines *et al*. [36] showed that Cav-1 is a positive regulator of both TGF-b and CTGF genes expression and signaling in human dermal fibroblasts and they found increased levels of Cav-1 in SSc fibroblasts.

The goal of this work was to evaluate whether an alterations in Cav-1 expression, affecting perivascular cells, may contribute to the pathogenic events leading to fibrosis during SSc, via the persistence of the VEGF signaling in the these cells. In this work, we provide evidence that a downregulation of Cav-1 in MSCs, isolated from SSc patients, strongly supports the pro-fibrotic effect of VEGF165, whose role in SSc has been largely explored [23-26]. In fact, an impairment of Cav-1 mediated internalization and degradation of VEGFR2 leads to a vicious loop, with redundant effects of VEGF signaling and consequent increased CTGF production. Furthermore, to better clarify the mechanisms linking the loss of Cav-1 to the abnormal persistent VEGF signaling in SSc, we silenced Cav-1 expression in HC-MSCs by specific siRNA, and observed that the decreased Cav-1 expression in HC-MSC is associated to a persisting VEGF signaling and CTGF production, mirroring the results observed in SSc-MSCs. These data confirm the lack of Cav-1 in perivascular cells may lead to an aberrant production of CTGF, which may contribute to the progression of fibrosis in SSc patients.

## Results

### Cav-1 levels are decreased in SSc-MSCs

**Figure 1:**
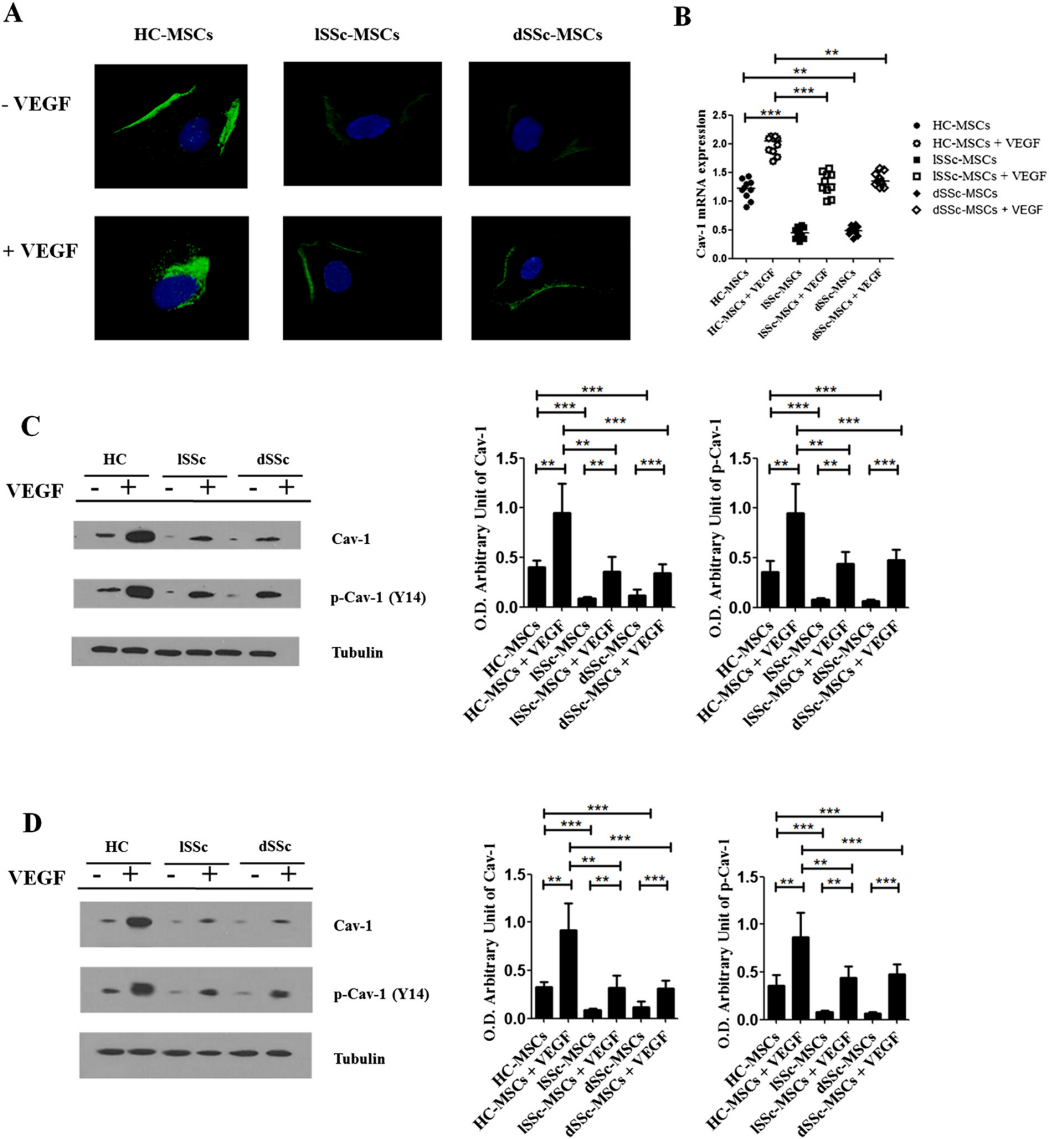
**Cav**-**1 modulation in SSc**-**MSCs. (A)** Immunofluorescence staining of Cav-1 in limited and diffused SSc-MSCs and HC-MSCs. The pictures showed an impairment of the Cav-1 trafficking in SSc cells. Pictures are representative of all experiments. Original magnification 20X. **(B)** Cav-1 mRNA levels of expression. After 15 min of VEGF treatment, the mRNA Cav-1 levels were markedly increased, in both HC- and SSc-MSCs, and the values were significantly higher in HC-MSCs *vs.* SSc-MSCs. Each experimental condition was performed in triplicate. All the results are expressed as Median (range) ***P <0.0001. **(C)** Western blot analysis of Cav-1 and Cav-1-phosphorilation (p-Cav-1) in HC- and SSc-MSCs, using neutral detergent. The western blot analysis confirmed the decrease of Cav-1 expression in SSc-MSCs and showed that VEGF induced a significant upregulation and a tyrosine phosphorylation of Cav-1, although the levels were significantly lower in SSc-MSCs when compared with HC cells. Each experimental condition was performed in triplicate. Blot was representative of all the experiments. The values were expressed as arbitrary unit of optical density (OD) (**P <0.001; ***P <0.0001). **(D)** Western blot analysis of Cav-1 and Cav-1-phosphorilation (p-Cav-1) in HC- and SSc-MSCs, using SDS. The results obtained by using a different buffer mirror those obtained by neutral detergent. The values were expressed as arbitrary unit of OD (**P <0.001; ***P <0.0001).

The immunofluorescence staining showed that in the unstimulated HC-MSCs, Cav-1 was primarily expressed in the surface of cells and after treatment with VEGF for 15 min, Cav-1 was internalized to the cytoplasm as shown by intracellular and perinuclear staining. On the contrary, an impairment of the Cav-1 trafficking was observed in the SSc cells, associated to a decreased surface expression of Cav-1, in both limited SSc (lSSc) and diffused (dSSc)-MSCs (Figure 1A).

Before VEGF treatment, in both lSSc- and dSSc-MSCs, independent from the duration of the disease, mRNA levels of Cav-1 were significantly lower when compared with basal HC-MSCs expression (Cav-1 mRNA levels: 0.45 (range, 0.34 to 0.58) in lSSc-MSCs and 0.49 (range, 0.34 to 0.60) in dSSc-MSCs, both *vs.* 1.21 (range, 0.89-1.43) in HC-MSCs, *P* <0.0001 and *P* = 0.0008, respectively). After 15 min of VEGF administration, the mRNA Cav-1 levels markedly increased in both SSc- and HC-MSCs (Cav-1 mRNA levels: 1.34 (range, 1.02 to 1.56) in lSSc-MSCs and 1.46 (range, 1.23 to 1.56) in dSSc-MSC, both *vs.* 2.04 (range, 1.70 to 2.13) in HC-MSCs, *P* <0.0001 and *P* = 0.0007, respectively) (Figure 1B). Western blot analyses of the proteins obtained with different buffers, confirmed that decreased production of Cav-1, in lSSc- as well as in dSSc-MSCs, confirming that, the treatment with VEGF induces, on one hand, a significant upregulation of Cav-1 and, on the other hand, its phosphorylation at Y14. Both the Cav-1 protein levels and its phosphorylation were significantly lower in SSc-MSCs when compared to those observed in HC cells (Figure 1C and 1D).

### The Cav-1 downregulation is associated with an impaired VEGFR2/Cav-1 co-localization in SSc-MSCs

**Figure 2:**
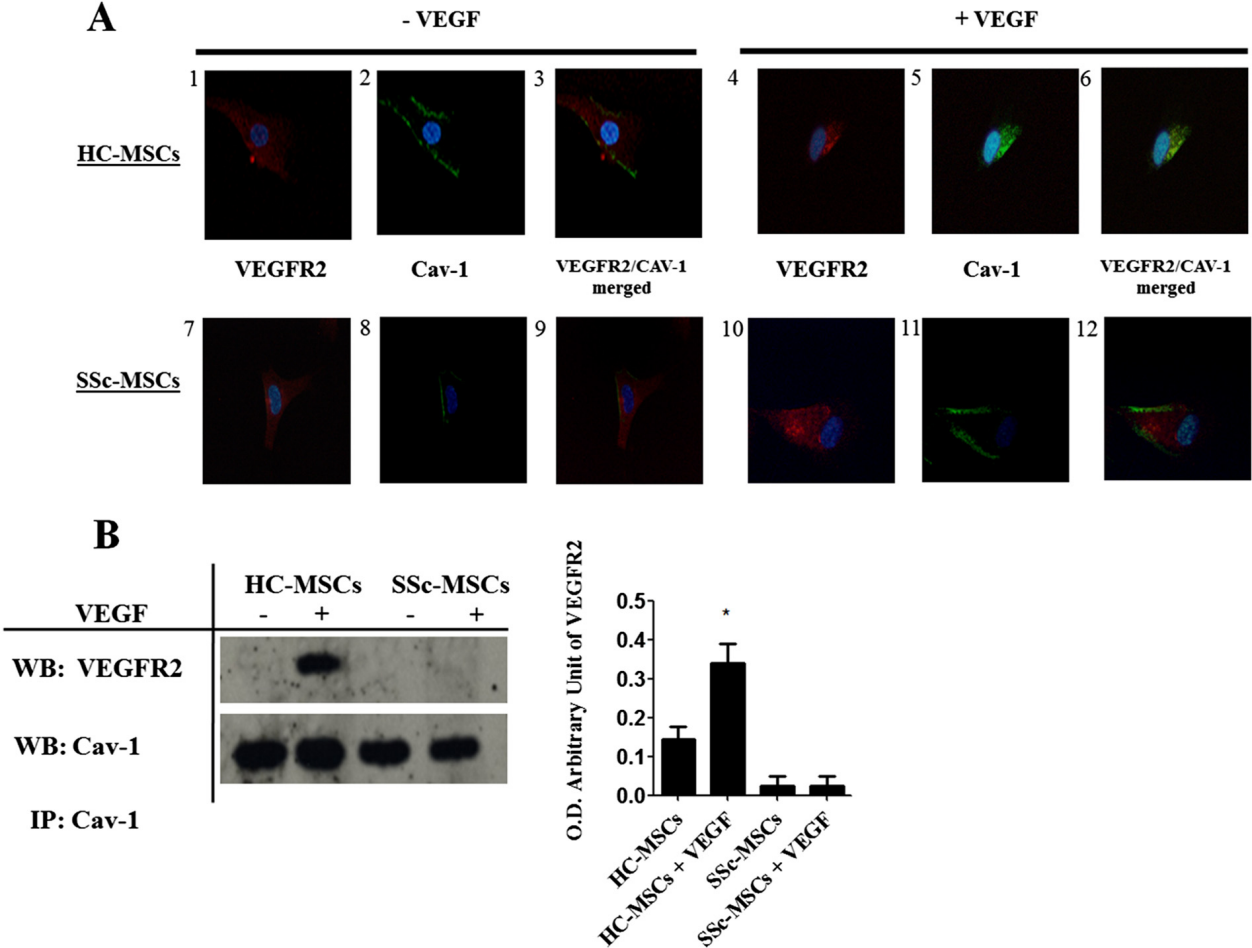
**Impaired Cav-1/VEGFR2 co-localization in SSc-MSCs. (A)** Immunofluorescence staining of Cav-1 (green) and VEGFR2 (red) in MSCs from HC and SSc. In unstimulated HC-MSCs, Cav-1 (2) is aggregated in some areas of the cell surface while the VEGFR2 (1) is ubiquitously diffuse. After treatment with VEGF both VEGFR2 and Cav-1 (4-6) were co-localized. In the SSc-MSCs, we observed an impaired VEGFR2/Cav-1 co-localization (7-12), after VEGF treatment. Pictures are representative of all experiments. Original magnification 20X. **(B)** Cav-1 was immunoprecipitated (IP) and its association with VEGFR2 was assessed by western blot (WB). The immunoprecipitation assay showed a lack of that Cav-1/VEGFR2 co-localization in SSc-MSCs. Each experimental condition was performed in triplicate. Blot was representative of all the experiments. Co-immunoprecipitated protein bands were quantified by densitometry and the values were expressed as arbitrary unit of optical density (OD) (**P <0.001).

By double staining immunofluorescence assay, after VEGF treatment, a strong VEGFR2/Cav-1 co-localization was observed in HC-MSCs. On the contrary, in the SSc-MSCs, a lack of VEGFR2/Cav-1 co-localization was observed (Figure 2A). To further explain these results an immunoprecipitation assay was performed, and our results are showed in Figure 2B. After VEGF treatment, VEGFR2 protein was detected in Cav-1 immunoprecipitates obtained from HC-MSCs protein extracts; on the contrary, in SSc-MSCs the Cav-1/VEGFR2 co-localization was absent.

### Reduced ubiquitination of VEGFR2 involve an increase of VEGF signaling in SSc-MSCs

**Figure 3:**
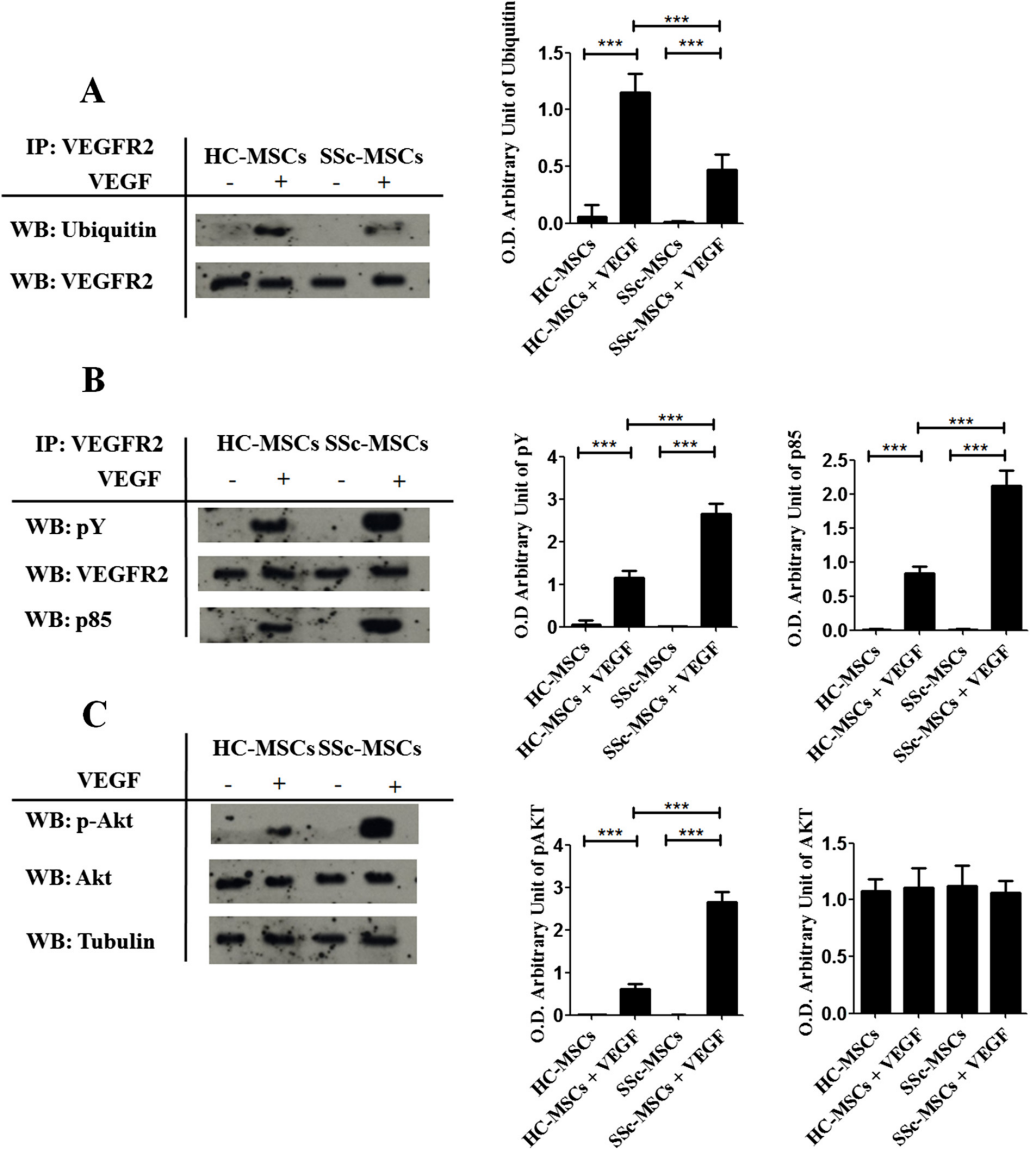
**Impaired ubiquitination of VEGFR2 and increased VEGF signaling in SSc**-**MSCs. (A)** VEGFR2 was immunoprecipitated (IP) and its association with ubiquitin was assessed by western blot (WB). The immunoprecipitation assay showed that in HC-MSCs, after VEGF treatment, the VEGFR2 immunoprecipitated complexes contained ubiquitin. In SSc-MSCs a decreased level of Ubiquitin associated to the VEGFR2 was observed. **(B)** Proteins immunoprecipitated (IP) with VEGFR2 antibodies were fractionated by SDS-polyacrylamide gel electrophoresis. Immunoblots were probed with an antibody to phosphotyrosine (pY) and p85. After VEGF treatment, the tyrosine phosphorylation of VEGFR2 in SSc-MSCs was markedly increased when compared to HC-MSC. **(C)** Western blot of phosphorylated Akt and total Akt. In SSc-MSCs, after VEGF treatment, the levels of p-Akt were higher when compared to HC. Each experimental condition was performed in triplicate. The blots in A-B-C were representative of all the experiments. Protein bands were quantified by densitometry and the values were expressed as arbitrary unit of optical density (OD) (***P <0.0001).

Unstimulated and stimulated MSCs, from both SSc patients and HC, were lysed and VEGFR2 was successively immunoprecipitated and analyzed by an anti-ubiquitin antibody, because of VEGFR2 should undergo ubiquitination before degradation. Our experiments showed that, in HC-MSCs, the VEGF receptor was ubiquitinated after VEGF administration, thus confirming that physiological degradation process started, after stimulation. On the contrary, in SSc-MSCs the levels of ubiquitin were significantly lower (Figure 3A), suggesting an impaired process of VEGFR2 degradation.

To confirm that, an impaired ubiquitination of VEGFR2 in SSc-MSCs, might modulate an increasing VEGF signaling, we investigated the PI3-kinase-Akt pathway, which has been reported to play a central role in VEGF activity [13]. We observed, by immunoblot analysis of VEGFR2 immunoprecipitates from HC-MSCs, that VEGF stimulation promoted the tyrosine phosphorylation of VEGFR2 and its interaction with PI3-kinase p85 subunit (Figure 3B). On the contrary, in SSc-MSCs, after VEGF treatment the tyrosine phosphorylation of VEGFR2 was markedly increased when compared to healthy cells. Furthermore, after VEGF treatment, we observed, by western blot, that the levels of phosphorylated Akt were significantly higher in SSc-MSCs when compared to HC-MSCs (Figure 3C).

### VEGF induces CTGF expression in SSc-MSCs

**Figure 4:**
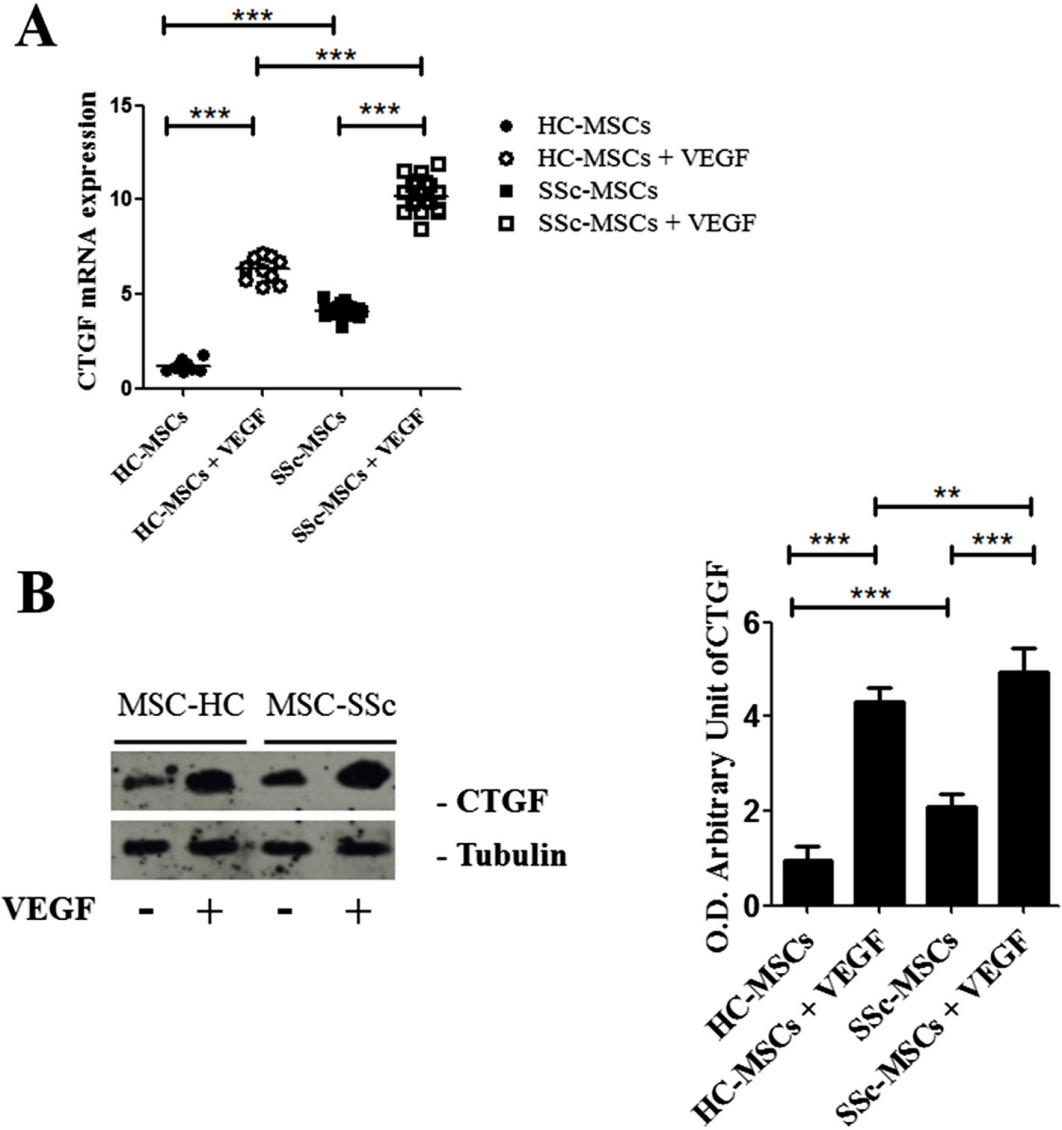
**CTGF expression in SSc**-**MSCs. (A)** qRT-PCR analysis of mRNA expression of CTGF, before and after VEGF treatment. Each experimental condition was performed in triplicate. All the results are expressed as Median (range) (***P <0.0001). **(B)** Western blot of CTGF expression. VEGF stimulation increased CTGF proteins levels in both SSc-ubiquitously and HC-MSCs, with a significantly higher expression in SSc-MSCs. Each experimental condition was performed in triplicate. Blot was representative of all the experiments. The values were expressed as arbitrary unit of optical density (OD) (**P <0.001; ***P <0.0001).

The CTGF gene expression was assessed by qRT-PCR. Before VEGF treatment, CTGF mRNA levels in both forms of SSc, independent of the disease duration, were higher than those observed in HC-MSCs (CTGF mRNA levels: 4.12 (range, 3.24 to 4.87) in SSc-MSCs *vs.* 1.16 (range, 0.88 to 1.78) in HC-MSCs, *P* <0.0001). The VEGF stimulation significantly increased the CTGF gene expression, both in HC- and SSc-MSCs, and of note, the CTGF mRNA levels of SSc-MSCs were again significantly higher than those observed in HC-MSCs (CTGF mRNA levels: 10.17 (range, 8.46 to 11.87) in SSc-MSCs *vs.* 6.32 (range, 5.35 to 7.19) levels in HC-MSCs, *P* <0.0001) (Figure 4A). The western blot analysis confirmed the mRNA results (Figure 4B).

### Increased VEGF signaling and CTGF production in HC-MSCs treated with Cav-1-siRNA

**Figure 5:**
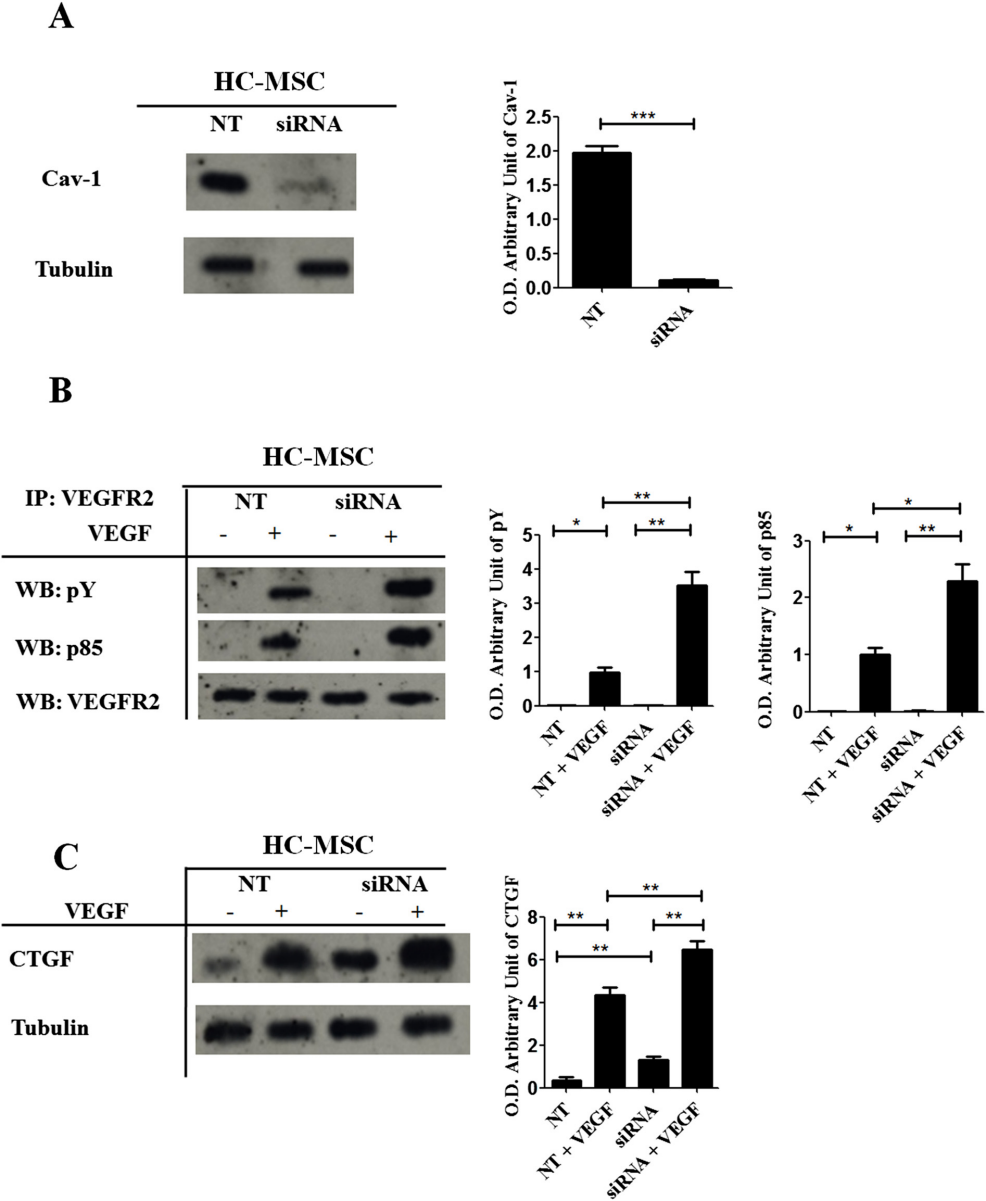
**Downregulation of Cav**-**1 in HC**-**MSCs by Cav**-**1**-**siRNA impaired VEGF signaling. (A)** HC-MSC was transfected with specific Cav-1-siRNA (siRNA) or non-targeting siRNA (NT), and Cav-1 protein expression was evaluated by western blot. The cells transfected with Cav-1-siRNA showed a decreased protein expression of Cav-1 when compared with cells transfected with NT siRNA. **(B)** Proteins immunoprecipitated (IP) by VEGFR2 antibodies were fractionated by SDS-polyacrylamide gel electrophoresis. The immunoblots were probed, by using specific antibodies against phosphotyrosine (pY) and p85. In siRNA HC-MSCs, after VEGF treatment, the tyrosine phosphorylation of VEGFR2 was significantly increased when compared to NT MSCs. **(C)** Western blot of CTGF expression. VEGF stimulation significantly increased CTGF proteins levels in siRNA HC-MSCs, when compared to NT HC-MSCs. Each experimental condition was performed in triplicate. Blot was representative of all the experiments. The values were expressed as arbitrary unit of optical density (OD) (*P <0.01; **P <0.001; ***P <0.0001).

To investigate the functional role of Cav-1 loss in HC-MSCs, we employed RNA interference. In Figure 5A, we showed that in Cav-1-siRNA treated HC-MSCs, a transient silencing of Cav-1 was observed, when compared to cells treated by negative non-targeting siRNA (NT). In Cav-1-siRNA treated HC-MSCs, the immunoblot analysis of VEGFR2 immunoprecipitates showed that VEGF stimulation promoted an increased tyrosine phosphorylation of the receptor and its interaction with PI3-kinase p85 subunit, whose levels were significantly increased when compared to NT HC-MSCs (Figure 5B). Furthermore, VEGF stimulation increased the CTGF gene expression, both in Cav-1-siRNA and NT treated HC-MSCs, although the CTGF protein expression of Cav-1-siRNA HC-MSCs was significantly higher than those observed in NT HC-MSCs (Figure 5C).

## Discussion

Fibrosis, characterized by excessive extracellular matrix accumulation, is the hallmark of SSc. Experimental studies suggest that a complex network including endothelial cells, pericytes, fibroblasts, and immune cells may lead to the pathogenic events of the disease. Many cytokines such as TGF-b, VEGF, CTGF, and endothelin-1 are considered as key mediators in this process, because of their skill to induce the differentiation of fibroblasts toward a myofibroblastoid phenotype [21,37].

A better understanding of the mechanisms that form an initial vascular alteration leading to a fibrotic response might suggest new potential targets for anti-fibrotic therapies of SSc. In this light, recent studies focused on the differentiantive ability of perivascular cells toward myofibroblast in fibrotic disorders. Dulauroy *et al*. [38], showed that the perivascular cells derived from mesenchymal lineage differentiated toward myofibroblast in an experimental model of kidney fibrosis. Furthermore, pericytes were shown to be the major source of myofibroblasts, thus opening new perspective about the role of perivascular cells in fibrosis.

In this work, using MSCs isolated from bone marrow of SSc patients, which are largely accepted as an alternative source of functional pericytes [11-15,18], we provide evidence that these cells display a failure in Cav-1 trafficking inside the cell, which lead to a significant decrease of VEGFR2 proteosomal degradation, upregulation of the VEGF downstream pathways, and finally, a consequent increased expression of CTGF. This abnormal persistent VEGF signaling in SSc perivascular mesenchymal cells, associated with an increased expression of CTGF, which is a pivotal cytokine involved in the development of tissue fibrosis, suggests that pericytes might be considered a new potential therapeutic target in SSc.

The VEGF signaling activation involves its binding to VEGFR2 and consequent internalization into the cytoplasm. The endocytosis of the receptor tyrosine kinases (RTKs) family may be divided into two different steps: the first step is internalization by translocation from the cell surface into intracellular vesicles. Internalization mainly controls the number of cell surface receptors and the sensitivity of the cell to incoming signal. In the second step, internalized receptors are sorted through a complex system of intracellular vesicle compartments, known as endosomes [39-41]. From early endosomes, which are characterized morphologically by small size and proximity to the plasma membrane [42], incoming ligand-receptor complexes are sorted into two different fates: recycling or degradation compartments. At present, it is well known that a dysfunctional regulation of RTKs endocytosis may promote uncontrolled activation of signaling [43].

Two different internalization pathways have been postulated for VEGFR2: the Caveolae-mediated endocytosis and the Clathrin-mediated internalization. The central question of whether or not VEGFR2 uses caveolar route for internalization cannot be unambiguously answered based on available data [44].

Caveolae are plasmalemmal caveolin-coated invaginations with trafficking functions, whose activity depends by the main protein Cav-1. Cav-1 oligomers constitute the filaments covering the cytosolic surface of caveolae [45]. These oligomers form a scaffold for the assembly of many signaling molecules, including receptors, their ligands, and signal transducers, thus regulating the activation and degradation state of these complexes [46].

Conflicting results have been published in available literature, concerning the possibility that VEGF may modulate the Cav-1 expression, probably due to the different cell types (endothelial, mesangial, fetoplacental artery endothelial cells) used in the experiments [47-49]. Furthermore, no data are available about the VEGFR2/Cav-1 interaction and its functions in MSCs. Normally, in the unstimulated state, caveolae are static structures, but after surface receptor stimulation, caveolae form active intracellular vesicles. Phosphorylation of Cav-1 (Y14) by Src kinase are required for caveolar fission [44]. The localization of both receptors and signaling partners to caveolae, has been considered a pivotal mechanism to control the levels of both receptors and signaling proteins, their availability, and activation.

In our work, we showed that in MSCs VEGF upregulates the levels of Cav-1 and its phosphorylation, suggesting that this mechanism may be involved in the following VEGFR2 activation. Conflicting results have been recently published concerning the optimal methods to evaluate the caveolae-associated Cav-1 [47,49,50]. Thus, in this paper, we lysed the cells by using neutral detergent, which completely dissolved the Cav-1 associated lipid micro-domains, or alternatively SDS, which did not detach the Cav-1 molecule from the caveolae. Independently of the constituents of buffer in SSc-MSCs stimulated and unstimulated by VEGF, the Cav-1 levels and its phosphorylation were always significantly lower when compared to HC-MSCs. Moreover, in untreated HC-MSCs, Cav-1 was primarily localized at the plasma membrane, and, after VEGF treatment, Cav-1 was internalized into the cytoplasm around the perinuclear area. On the contrary, this vesicular trafficking was significantly impaired in SSc-MSCs.

To assess the pathogenetic implication of the reduced Cav-1 expression in the specific trafficking and signaling of VEGFR2 during SSc, we evaluated the Cav-1/VEGFR2 co-localization, after VEGF stimulus. Our data showed that in SSc-MSCs this molecular co-localization was significantly impaired, when compared to HC-MSC and these data were confirmed by IF and immunoprecipitation.

The signaling of VEGF starts after VEGFR2 internalization and the fate of activated receptors depends on its transport to late endosome for degradation, or alternatively for recycling. Several studies, carried out on different RTKs suggest that the transport to late endosome should be mediated by Cav-1 [51,52] and a decrease of Cav1 may result in enhanced growth factor signaling [51]. The receptor degradation occurs in the proteasome. It recognizes and rapidly degrades ubiquitinated proteins involved in the regulation of transcription, cell cycle progression, apoptosis, signal transduction, and angiogenesis [53,54]. Ubiquitin is a conserved 76-amino-acid polypeptide, activated by an ubiquitin-activating enzyme (E1) in an ATP-dependent manner and it is transferred to an ubiquitin-conjugating enzyme (E2). Furthermore, an ubiquitin-protein ligase (E3) specifically attaches ubiquitin to a target protein by an amino group of a lysine residue. E3 ubiquitin ligases are a large family of proteins that are known to be involved in the regulation of turnover and activity of many target proteins. Although it has been established that VEGFR2 is ubiquitinated in a ligand-dependent manner in response to VEGF [53], the role of ubiquitination in VEGF signaling is still unclear. Several ubiquitin ligases are known to be involved in VEGFR2 degradation and attenuation of signaling [55]. Earlier studies established that an E3 ubiquitin ligase, Nedd4, modulates the proteasomal degradation of VEGFR2, and that this degradation might be inhibited by the sequestration of Nedd4 [54]. It may be considered that the amount of ubiquinated VEGFR2, co-localizing with Cav-1 [33,55-57] may be carried to degradation, following the same fate of other RTKs [44]. We observed that the levels of ubiquitinated VEGFR2, after VEGF treatment, were significantly reduced in SSc-MSC and this decreased level of ubiquitination might contribute to upregulation of VEGF signaling. In fact, different studies showed that ubiquitin-mediated proteolysis plays an important role in the regulation of many cellular processes, including receptors activity, by facilitating the timely destruction of key proteins [58-60]. It is well known that VEGF may induce CTGF production [59], and in this setting we observed that following the increased VEGF signaling in SSc-MSCs, a consequent increased expression of CTGF may be shown. This molecule, also known as CCN2, is a member of the CCN (CCN1-6) family of matricellular proteins. Many studies focused on the role of CTGF in increasing ECM production, during fibrosis [61]. CTGF is generally overexpressed in all fibrotic conditions, and induces collagen type I deposition [62] and it has been suggested to play a crucial role in SSc tissue fibrosis [61-64]. It has been shown to induce fibroblast proliferation, cell adhesion, and stimulation of extracellular matrix production [65]. it has been reported that VEGF is able to modulate CTGF production [21], in fact, VEGF may affect both the proliferation of fibrocellular components and the wound healing process, via CTGF induction [19,20]. Interestingly, the SSc-MSCs production of CTGF was significantly higher when compared with healthy cells and the increased levels of VEGF observed in SSc patients [23] may contribute to the over expression of CTGF, which plays different physiological roles, not only in fibrotic process but also in angiogenesis, such as the maintenance of capillary strength via the extracellular matrix production [66,67].

To confirm the functional role of loss of Cav-1 in VEGF signaling and CTGF modulation, we silenced the Cav-1 expression in HC-MSCs. We showed that the downregulation of Cav-1 induced a significant increase in VEGFR2 phosphorylation and its interaction with PI3-kinase p85 subunit, which is associated to an increase of VEGF signaling, in knockdown cells, when compared to NT HC-MSCs, thus mirroring the same results observed in SSc-MSCs.

This Cav-1 downregulation further strengthens our data in SSc patients, and supports the hypothesis that the loss of Cav-1 in SSc-MSCs may be involved in the molecular pathogenetic steps of the disease, and linking the earlier vascular damages to the subsequent fibrosis.

## Conclusion

This is the first paper, in our knowledge evaluating the function of Cav-1 on the VEGF signaling in SSc, and particularly on MSC/pericyte lineage [1], showing that these pathways may strongly influence fibrotic process during scleroderma. These data underline the potential role of Cav-1 in fibrotic diseases and suggest this molecule as a possible therapeutic target in the disease. Furthermore, the evidence of an impaired expression and function of Cav-1 in perivascular SSc cells strongly confirms the potential role of the MSCs/pericytes in this pathologic process probably in the earlier phases of the disease following the initial endothelial injury. Taken together our data suggest that MSCs/pericytes lineage, localized in the perivascular niches, may be a potential cellular target in SSc and open new perspectives in the field of MSC transplantation and regenerative medicine in scleroderma.

## Methods

### Isolation, culture, immunophenotyping, and differentiation of MSCs

After approval of San Salvatore University Hospital ethics committee and written informed consent from patients, primary MSCs were obtained from 20 SSc patients, 10 lSSc, and 10 dSSc by aspiration from the posterior superior iliac crest. Demographic and clinical characteristics of the patients are showed in Table 1. Patients discontinued corticosteroids, oral vasodilators, intravenous prostanoids, or other potentially disease-modifying drugs, at least 1 month before biopsies. None assumed immunosuppressants. Ten frozen BM-MSCs samples obtained from age-matched healthy women bone marrow donors were used as control. Both SSc- and HC-MSCs were cultured and characterized as previously described [33]: plated at concentration of 2 × 10^5^ cells/cm^2^ in Dulbecco’s Mod Eagle Medium (D-MEM, GIBCO, Carsbald, CA, USA), supplemented with 10% fetal bovine serum (Standard South America origin, Lonza, Walkersville, MD, USA), 2 mmol/L L-glutamine (EuroClone, Milan, Italy), and 100 U Penicillin, 1,000 U Streptomycin (Biochrom AG, Miramar, FL, USA). At 80% confluence the MSCs were split and subcultured.

**Table 1:** Clinical and demographic features of the 20 SSc patients

**Sex/Age (years)**	**Year of SSc onset**	**MRSS**	**Autoantibodies**	**Lung involvment HRCT/PFT**	**Heart and kidney involvement**	**Digital ulcers**
F/22	2009	14	ANA/ACA	Normal/Normal	Normal/Normal	Yes
F/45	1998	13	ANA/ACA	Normal/Normal	Normal/Normal	Yes
M/43	1985	18	ANA/ACA	Normal/Normal	Normal/Normal	Yes
F/26	2003	8	ANA/ACA	Normal/Normal	Normal/Normal	Yes
F/39	1980	15	ANA/ACA	Normal/Normal	Normal/Normal	No
F/40	2002	8	ANA/ACA	Fibrosis/Normal	Normal/Normal	Yes
M/45	2000	10	ANA/ACA	Normal/Normal	Normal/Normal	Yes
F/21	2010	13	ANA/ACA	Normal/Normal	Normal/Normal	Yes
F/30	2000	9	ANA/ACA	Fibrosis/Normal	Normal/Normal	Yes
F/22	2008	11	ANA/ACA	Normal/Normal	Normal/Normal	Yes
F/40	1978	12	ANA/Scl-70	Normal/Normal	Normal/Normal	No
F/41	2001	10	ANA/Scl-70	Fibrosis/Normal	Normal/Normal	Yes
F/23	2007	13	ANA/Scl-70	Normal/Normal	Normal/Normal	Yes
F/46	2007	12	ANA/Scl-70	Normal/Normal	Normal/Normal	No
F/21	2007	13	ANA/Scl-70	Normal/Normal	Normal/Normal	Yes
F/31	2000	15	ANA/Scl-70	Normal/Normal	Normal/Normal	Yes
F/36	1999	20	ANA/Scl-70	Normal/Normal	Normal/Normal	Yes
F/20	2008	11	ANA/Scl-70	Normal/Normal	Normal/Normal	Yes
F/41	2004	20	ANA/Scl-70	Normal/Normal	Normal/Normal	Yes
F/30	2007	10	ANA/Scl-70	Normal/Normal	Normal/Normal	Yes

ACA = anti-centromere antibodies ANA = antinuclear antibodies; HRCT = high resolution computed tomography; MRSS = modified Rodnan skin thickness score (maximum possible score 51); PFT = pulmonary function test.

Third-passage (P3) MSCs were analyzed for the surface expression of mesenchymal antigens (CD45, CD73, CD90, CD34, CD79a, PDGFRβ) and pericyte markers (α-SMA, SM22α, NG2, desmin, RGS5) as previously described [33].

### MSCs treatment with VEGF

To establish the optimal concentration of VEGF_165_ (R & D, USA), in our system, a dose/response curve was performed, using P3 MSCs obtained from both one control and one patient.

Each experiment was performed in triplicate and the optimal stimulation dose for VEGF was always assessed to be 50 ng/mL.

### Immunofluorescence

Cells were placed in eight wells culture slides (BD, USA) and treated with VEGF 50 ng/mL for 15 min. Control cells were cultured in basal medium. For the staying, the cells were incubated with Cav-1 and VEGFR2 antibodies (Santa Cruz Biotechnology, CA, USA). After, the cells were incubated with secondary antibodies (Alexa Fluor 488-conjugated and Alexa Fluor 555-conjugated, Invitrogen, USA) and counterstained using 4′, 6-diamidino-2-phenylindole (DAPI). Images were acquired using an Olympus BX53 fluorescence microscope.

### qRT-PCR analysis

Total RNA was extracted from VEGF treated and untreated MSCs using TRIZOL (SIGMA, USA) and reverse transcribed into complementary DNA (cDNA) with the ThermoScript reverse transcription-PCR system (Invitrogen, CA, USA). The qRT-PCR was performed by using SYBR green kits (Applied Biosystems, The Netherlands). Results were analyzed after 45 cycles of amplification using the ABI 7500 Fast Real Time PCR System. Primers were designed on the basis of the reported sequences (Primer bank NCBI; CTGF: 5′-CAGCATGGACGTTCGTCTG-3′ (forward) and 5′-AACCACGGTTTGGTCCTTGG-3′ (reverse); Cav-1: 5′-AATACTGGTTTTACCGCTTGCT-3′ (forward) and 5′-CATGGTACAACTGCCCAGATG-3′ (reverse); β-actin: 5′-CCTGGCACCCAGCACAAT-3′ (forward) and 5′-AGTACTCCGTGTGGATCGGC-3′ (reverse)). The qRT-PCR was run in triplicate.

### RNA interference

For silencing of Cav-1 expression, HC-MSCs were transfected with Silencer Select Cav-1-siRNA (Life Technologies, USA) or with Silencer Select Negative Control non-targeting siRNA (NT) (Life Technologies, USA) using Lipofectamine™ 3000 (Life Technologies, USA). Transfection was performed according to the manufacturer’s instructions. Briefly, HC-MSCs were plated at 1 × 10^4^ cells per cm^2^, 24 h prior to transfection. Cultures were incubated for 24 h with 25 pmol of siRNA in 2 mL of OptiMem. After incubation, plates were washed and cells were allowed to recover in normal growth conditions (10% DMEM) for 24 h post-transfection.

### Western blot

In order to perform western blot assays, HC- and SSc-MSCs, before and after treatment, were pelleted, washed twice with PBS, lysed on ice in lysis buffer (1% Triton X-100, 0.5% NP-40, 50 mM Tris-Cl, pH 7.5, 150 mM NaCl, 1 mM EDTA, supplemented with 1 mM phenylmethylsulfonyl fluoride, 1 mM NaF, 1 mM Na_3_VO_4,_ 5 μg/mL aprotinin, 5 μg/mL leupeptin) for 30 min, and cleared by centrifugation [49]. Due to the reported data that neutral detergent, such as Triton X-100, does not identify the amount of caveolae-associated Cav-1 [50], six SSc-MSCs (3 lSSc and 3 dSSc) and three HC-MSCs, before and after treatment, were pelleted, washed twice with PBS, solubilized with a different sample buffer, containing 0.125 M Tris-HCl (pH 6.8), 5% (w/v) SDS, 2.5% (v/v) b-mercaptoethanol, 5% glycerol in double distilled water [47].

The protein concentration was calculated by Bradford protein assay reagent (Bio-Rad). A total of 50 μg of proteins were separated by SDS-PAGE and transferred to nitrocellulose membranes. After 1 h blocking at room temperature in blocking buffer (5% not fat milk in Tris-buffered saline/1% tween 20 (TBS/T)) and after washing three times for 5 min each in TBS/T, the membranes were incubated overnight at 4°C with the primary antibodies: Cav-1 and phospho-Cav-1 (Y14) (Santa Cruz Biotechnology, CA, USA), phospho-Akt and Akt (New England Biolabs, MA, USA) and CTGF (R & D Systems, MN, USA), diluted in 5% bovine serum albumin in TBS/T. Following three washes with TBS/T, horseradish peroxidase-conjugated secondary antibodies (Santa Cruz, Biotechnology, CA, USA) diluted in blocking buffer was added for 30 min at room temperature and washed three times with TBS/T. The detection was performed by enhanced chemiluminescence detection ECL reaction (Amersham Pharmacia Biotechnology). Tubulin signal (CP06 Anti-α-Tubulin Mouse mAb-DM1A) was used as loading control. Immunoreactive bands were quantified with densitometry using ImageJ software (NIH, Bethesda, MD, USA).

### Immunoprecipitation

Cells were washed three times with cold phosphate-buffered saline and solubilized in 200 μL of lysis buffer (TrisHCl 10 mM, ph 7,4, NaCl 100 mM, EDTA 1 mM, EGTA 1 mM, Triton 1%, NP-40 0,5%, NaF 50 mM, Na3VO4, PMSF 1 mM, Leupeptin 10 μg/mL, Aprotinin 10 μg/mL).

After centrifugation at 12,000 rpm for 10 min, 0.5 mg of protein was subjected to immunoprecipitation. Specific rabbit anti-Cav-1 and anti-VEGFR2 antibodies (Santa Cruz Biotechnology, CA, USA) was added and rocked at 4°C for 1 h; 30 μL protein A/G beads (Santa Cruz) was added and the sample was rocked over night at 4°C.

For western blotting, anti-VEGFR2 (Santa Cruz Biotechnology, CA, USA), anti-Cav-1 (Santa Cruz Biotechnology, CA, USA), anti-ubiquitin, anti-p85, and anti-phosphotyrosine (Abcam, MA, USA) were used.

### Statistical analysis

GraphPad Prism 5.0 software were used for statistical analyses. Where not differently specified, our results are expressed as Median (range), within a confidence interval of 95%. Due to the non-parametric distribution of our data the Mann-Whitney U test was used as appropriate for analyses. Statistical significance was expressed by a *P* value <0.05.

## Abbreviations

Cav-1: Caveolin-1; CTGF: Connective tissue growth factor; dSSc: diffused systemic sclerosis; ECM: Extracellular matrix protein; EGFR: Epidermal growth factor receptors; lSSc: Limited systemic slerosis; MSCs: Mesenchymal stem cells; RTKs: Receptor tyrosine kinases; SSc: Systemic sclerosis; TGF-b: Transforming growth factor-b; VEGF: Vascular endothelial growth factor; VEGFR2: Vascular endothelial growth factor receptor 2.

## Competing interests

The authors declare that they have no competing interests.

## Authors’ contributions

PC: study conception and design, data interpretation, literature search, figure creation, writing, paper revision, and acceptance; PDB: study conception and design, data interpretation, literature search, figure creation, writing, paper revision, and acceptance; DC: data collection, literature search, paper revision, and acceptance; FZ: data collection, literature search, paper revision and acceptance; VL: data collection, literature search, paper revision, and acceptance; PR: data collection, data interpretation, literature search, paper revision, and acceptance; IP: data collection, literature search, paper revision, and acceptance; OB: data collection, literature search, paper revision, and acceptance; FC: data collection, data interpretation, literature search, paper revision, and acceptance; EA: data collection, data interpretation, literature search, paper revision, and acceptance; RG: study design, data interpretation, writing, paper revision, and acceptance. All authors gave final approval for submitting the manuscript for review and agree to be accountable for all aspects of the work.
